# The Role of Heredity and the Prevalence of Strabismus in Families with Accommodative, Partial Accommodative, and Infantile Esotropia

**DOI:** 10.4274/tjo.galenos.2019.49204

**Published:** 2020-06-27

**Authors:** Fatma Çorak Eroğlu, Sibel Oto, Feride İffet Şahin, Yunus Terzi, Özge Özer Kaya, Mustafa Agah Tekindal

**Affiliations:** 1 Ulucanlar Eye Training and Research Hospital, Ankara, Turkey; 2 Başkent University Faculty of Medicine, Department of Ophthalmology, Ankara, Turkey; 3 Başkent University Faculty of Medicine, Department of Medical Genetics, Ankara, Turkey; 4 Tepecik Training and Research Hospital, Genetic Diagnostic Center, İzmir, Turkey; 5 Selçuk University Faculty of Medicine, Department of Statistics, Konya, Turkey

**Keywords:** Strabismus, genetics, esotropia, inheritance

## Abstract

**Objectives:**

To investigate the prevalence of strabismus in families of a proband with accommodative, partial accommodative, or infantile esotropia (IET), and to evaluate the mode of inheritance and the role of consanguineous marriages in this prevalence.

**Materials and Methods:**

Families of probands with comitant strabismus were invited to participate in the study. The family members of 139 subjects with accommodative esotropia (AET), 55 with partial accommodative esotropia (PAET), and 21 with IET agreed to participate. Detailed family trees were constructed. The first- and second-degree relatives were invited for a complete ophthalmological examination, and 518 individuals from 168 families were evaluated. The role of consanguinity, the presence of tropia, phoria (≥8 PD), microtropia, and hypermetropia (≥3.00 D) among first- and second-degree relatives were analyzed.

**Results:**

A non-Mendelian pattern was found in 49 families (23%), an autosomal dominant pattern in 39 families (18%), and an autosomal recessive pattern in 6 families (3%). The prevalence of consanguineous marriages among parents of probands was 18.1%, 22.6%, and 14.3% in the AET, PAET, and IET groups, respectively (p=0.652). The prevalence of strabismus in first-degree relatives was 58.9%, 45.5%, and 38.1%, respectively (p=0.07). The prevalence of microtropia in probands’ siblings was significantly higher in the AET group (p=0.034).

**Conclusion:**

Sporadic cases and non-Mendelian inheritance were more frequent than autosomal recessive inheritance. Autosomal recessive inheritance was found not to be frequent in consanguineous marriages. The prevalence of strabismus and microtropia was significantly higher in families of esotropia cases than in the general population.

## Introduction

Comitant strabismus is a multifactorial disease with genetic and environmental components, in which the influence of environmental factors appears dependent on genetic susceptibility.^1,2,3^ The increased risk of strabismus among those with a family history of the condition has been known since the time of Hippocrates (470-360 BC), 2400 years ago.^4,5^ Although the strabismus rate in the general population is 2%-6%, several studies found that this rate varies from 13%-65% among families of affected individuals.^1,6,7,8,9,10^ Parikh et al.^11^ reported that having a first-degree relative with strabismus led to a 3-5-fold increase in the risk of developing the same condition, while investigation of a cohort of 7100 strabismus patients in 12 studies revealed that 30.6% of strabismic probands had a close relative with strabismus.^6^

Numerous modes of inheritance have been suggested for comitant strabismus, but none has been proven.^9,12,13,14^ Maumenee et al.^7^ suggested an autosomal recessive inheritance pattern for 173 pedigrees with infantile esotropia (IET), involving a total of 1589 family members. However, computer segregation analysis by the authors was most compatible with the presence of codominant genes. They therefore proposed that the disease best fitted a model of either multifactorial inheritance or codominant genes with incomplete penetrance.

Previous studies have assessed inheritance in comitant strabismus by performing genome-wide linkage scans and revealing a number of susceptibility loci.^11,15,16^ The present study evaluated the prevalence of strabismus in families with IET, partial accommodative esotropia (PAET), and accommodative esotropia (AET), with the aim of determining the mode of inheritance and the role of consanguinity in the heritability of different types of esotropia. We also investigated the frequency of coexisting visual impairments and the presence of hypermetropia of ≥+3.00 diopter (D) within these families.

## Materials and Methods

### Participants

This study was approved by the Başkent University Institutional Review Board and Ethics Committee (project no: KA 09/246) and was supported by Başkent University Research Fund (Ankara/Turkey). Written informed consent was obtained from all patients.

Two different strategies were applied to collect patient data. The first was a retrospective cross-sectional review of the medical records of all patients diagnosed with comitant esotropia at Başkent University Department of Ophthalmology between 1998 and 2010. A total of 215 families of probands agreed to participate in the study: 139 had a proband with AET, 55 with PAET, and 21 with IET. The following exclusion criteria were applied after detailed questioning: prematurity, organic amblyopia, presence of a neurodevelopmental disorder, residence outside the city, and failure to communicate with all members of a given family. A total of 518 individuals from 168 families then underwent ophthalmological examination. These included 116 AET families, 39 PAET families, and 13 IET families.

The second part of the study was conducted prospectively at Başkent University Department of Ophthalmology between June 2010 and August 2011.

### Ophthalmic examination

All 518 individuals underwent full ophthalmic examination by the same ophthalmologist (F.C.E.). The presence of deviation was determined using a prism cover test performed at distance and near fixation, with and without correction. The Worth four-dot test at distance, Randot stereopsis test at near, and 4-prism base-out test at distance were performed to evaluate binocular function. All tropias, microtropias, phorias ≥ +3.00 PD, hypermetropias >+3.00 D, and anisometropia were acknowledged as important clinical findings. Anisometropia was defined as unequal refractive error (with a difference in refractive error between the eyes of 2 D or more).

### Data Analysis

The parents of probands were interviewed to identify relatives with a history of strabismus, and detailed family trees were constructed. Each set of parents answered questions about the presence of consanguinity, high refractive errors, amblyopia, night blindness, and any other known eye disease among family members. Family trees were interpreted by members of the Department of Genetics and analyzed using Cyrilic 3 pedigree software (AP Benson, London, UK) to determine the mode of inheritance. Data were collected for 3 main aspects: the frequency of consanguinity among parents of probands; the frequency of strabismus among first-, second-, and third-degree relatives; and the frequency of hypermetropia ≥+3.00 D and anisometropia among first-degree relatives.

### Statistical Analysis

Categorical variables were statistically evaluated using Pearson’s χ^2^ test and the likelihood ratio χ^2^ test to reveal relationships between the variables; odds ratios were calculated for all risk factors identified. The likelihood ratio test was used because some cells of the contingency tables included values of zero or small frequencies. Data analyses were performed using SPSS software, version 17.0 (SPSS Inc., Chicago, IL). A p-value less than 0.05 was considered statistically significant.

## Results

**Figure 1:**
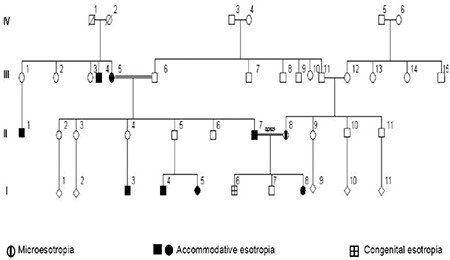
Autosomal recessive inheritance pedigree pattern with 3 affected generations

**Figure 2:**
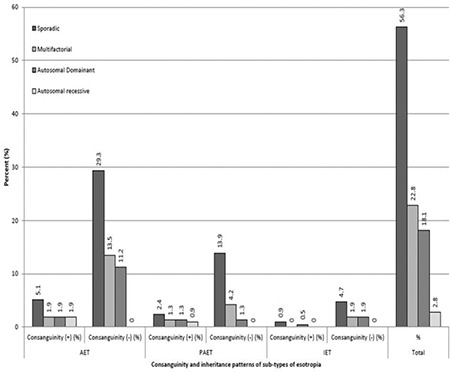
Distribution of consanguinity and inheritance patterns in esotropia subtypes ET: Esotropia, AET: Accommodative esotropia, PAET: Partial accommodative esotropia, IET: Infantile esotropia, (+): Presence of consanguinity, (-): Absence of consanguinity

Demographic properties of the study group are summarized in Table 1. Based on pedigree analysis, no definite mode of inheritance could be assigned to 121 families (56.3%), so the strabismic individuals in these families were considered sporadic cases. A non-Mendelian trait was found in 49 families (23%), an autosomal dominant pattern in 39 families (18%), and an autosomal recessive pattern in 6 families (3%). A sample of autosomal recessive inheritance pedigree pattern with affected 3 generations is shown in Figure 1. First-cousin marriage among parents of the proband was found in 20 subjects (16.5%) in the AET group, 11 (20.0%) in the PAET group, and 1 (4.8%) in the IET group. A history of second-cousin marriage was reported for 3 subjects (2.6%) in the AET group, 2 (2.6%) in the PAET group, and 2 (9.5%) in the IET group. There was no significant relationship between either the frequency (p=0.457) or degree (p=0.125) of cross-cousin (parents are opposite gender siblings) marriage among any of the esotropia subtypes studied. The distribution of consanguinity and established inheritance patterns for various sub-types of esotropia is shown in Figure 2. An autosomal recessive inheritance pattern was detected in 6 families, all of which reported consanguineous marriages; however, most consanguineous families displayed a sporadic mode of inheritance. There was no significant relationship between any esotropia subtype and the inheritance pattern suggested by pedigree analysis (p=0.682).

Examination of the pedigrees showed that the prevalence of strabismus in any first-degree relative of the proband was 54% for the group overall, 59% for the AET group, 45.5% for the PAET group, and 38.1% for the IET group. No significant difference was found between the three groups (p=0.077). The likelihood of having one parent with strabismus was 30.6% for the group overall, 35.3% for AET, 20% for PAET, and 28.6% for the IET group (p=0.113). Table 2 presents the prevalence and type of strabismus found parents and probands by ophthalmic examination. Based on ophthalmic examination, no correlation was found between the esotropia subtypes of probands and the prevalence of strabismus in their mothers (p=0.462). Notably, the fathers of 31 probands (26.7%) in the AET group and 5 (12.8%) in the PAET group had strabismus, while none of the fathers in the IET group was strabismic. The prevalence of strabismus in fathers was significantly higher in the AET group (p=0.027) compared with other groups. The OR for the increased likelihood of strabismus in AET compared with PAET proband fathers was 2.87 (95% confidence interval [CI]: 1.054-7.820).

Microtropia was the most prevalent deviation seen in mothers among all groups. However there was no significant relationship between the proband esotropia subtype and the type of strabismus in the mother (p=0.974) or the frequency of an esotropia subtype (p=0.914) and microtropia (p=0.852) found in the mother. Similarly, no significant relationship was observed between the esotropia subtype of the proband and the types of tropia (p=0.240) or subtypes of esotropia (p=0.219) observed in the examined fathers. Nonetheless, the frequency of microtropia was significantly higher among the fathers of probands with AET (p=0.046) compared with other groups.

**Figure 3:**
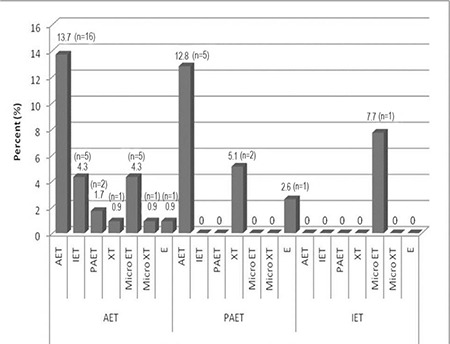
Types of strabismus found in siblings by examination Siblings of subjects in the AET group (p=0.012) were significantly more likely to have AET (p=0.047) or microtropia (p=0.034) than those in other groups AET: Accommodative esotropia, IET: Infantile esotropia, PAET: Partial accommodative esotropia, XT: Exotropia, MicroET: Microesotropia, MicroXT: Microexotropia, E: Esophoria

Figure 3 presents the types of strabismus found in affected siblings. The siblings of subjects in the AET group were significantly more likely to have AET (p=0.047) or microtropia (p=0.034) than the other groups. Siblings of those patients with AET were 3.0-fold (95% CI 1.102-7.671) more likely to develop strabismus relative to siblings of those with PAET. The number of affected siblings for each proband was also investigated. In the AET group, 29 probands had a total of 34 affected siblings. One proband had 2 affected siblings and 2 probands had 3 affected siblings. In the PAET group, 6 probands had a total of 8 affected siblings, and 1 proband had 3 affected siblings. In the IET group, 2 probands had 1 affected sibling.

Twenty-five first-degree relatives (1 mother, 5 fathers, 19 siblings) associated with 20 probands in the AET group had ≥3.00 D hypermetropia. In the PAET group, 2 probands had 1 first-degree relative with ≥3.00 D hypermetropia, and in the IET group, the mother of 1 proband had ≥+3.00 D hypermetropia. There was no significant relationship between the frequency of ≥+3.00 D hypermetropia among first-degree relatives and any particular esotropia subtype (p=0.113). Also, we found 9 myopic (4 mothers, 2 fathers, 3 siblings), 7 hypermetropic (2 mothers, 4 siblings), and 2 astigmatic (1 mother and 1 sibling) anisometropia in the AET group. In the PAET group, 3 myopic (2 mothers, 1 sibling) and 1 hypermetropic (1 mother) anisometropia, and in the IET group, the sibling of the 1 proband had hypermetropic anisometropia. Anisometropia was more frequent in mothers of AET and PAET groups but there was no significant relationship between the frequency of anisometropia among first-degree relatives and any particular esotropia subtype (p=0.324).

We also investigated coexisting ocular pathologies found in family members. In the AET group, 2 relatives had degenerative myopia, 1 relative had iridocyclitis, 3 relatives had keratoconus, and 2 relatives had primary open-angle glaucoma. In the PAET group, 1 relative had iridocyclitis and 1 relative had keratoconus. In the IET group, none of examined relatives displayed any comorbidity.

Based on the pedigree analysis of 215 strabismus cases included in the study, 43 probands (20.5%) had at least 1 second-degree relative who was strabismic; 54 (25.1%) had 1 third-degree relative with strabismus. There was no significant correlation between any given esotropia subtype and the frequency of strabismus observed among second- (p=0.193) or third-degree relatives (p=0.065).

## Discussion

The present study analyzed the roles of heredity and consanguinity in the development of strabismus by studying the frequency of various types of strabismic deviations among the families of the probands. In cases with cross-cousin marriages, multifactorial patterns of inheritance were more frequent than recessive modes of inheritance. Strabismus and microtropia were also significantly more prevalent among first-degree relatives and other family members compared with the general population.

For each esotropia subgroup, most cases (53.2% in the AET group, 63.6% in the PAET group, and 57.1% in the IET group) were sporadic. The pattern of inheritance was not compatible with a Mendelian trait, so the etiology was assumed to be polygenic or multifactorial. An autosomal dominant origin was found in 18.1% of cases. Although the frequency of consanguinity in AET, PAET, and IET groups was 16.5%, 23.6%, and 14.3%, respectively, an autosomal recessive mode of inheritance was only observed in 2.8% of all cases. Each of the autosomal recessive cases observed was associated with a cross-cousin marriage, but the families that included the offspring of a cross-cousin marriage exhibited mainly sporadic or multifactorial inheritance patterns. Articles reporting risk factors including study type, risk factors assessed, inheritance type, and significant findings are summarized in Table 3.^7,12,13,16,19,20,21,22,23,24,25,26^ Maconachie et al.^17^ made a systematic review of the literature relating to the risk factors and inheritance of comitant strabismus, and reported that most of the studies proposed a polygenic inheritance where genetic and environmental factors are involved. Family studies highlighted difficulties in assessing inheritance patterns for comitant strabismus because the patterns were not compatible with simple Mendelian models.

Bagheri et al.^18^ previously investigated the role of consanguinity as a risk factor for developing comitant strabismus. Their study included 461 patients categorized into 4 groups as exotropia (XT), IET, non-accommodative acquired esotropia, and accommodative acquired esotropia. These patients were compared with a control group of 421 healthy children. The rate of first-cousin marriage was 37.7% in the patient group and 23.5% in the control group. Following the calculation of inbreeding coefficients, the authors suggested that patients with non-accommodative acquired esotropia had the highest mean of inbreeding coefficient and recessive form of inheritance had an important role in the etiology of comitant strabismus.

Chaudhuri et al.^19^ prospectively evaluated consecutive families with 2 or more affected subjects with comitant horizontal strabismus. These included 18 families with esotropia and 18 families with XT. They found vertical transmission in 76.5% of families with XT and 54.54% of families with esotropia with significant familial concordance, the transmission was from the maternal side of the family.

A study by Richter^12^ of 697 probands with either eso- or XT and their available relatives suggested that a multifactorial etiology underlied the inheritance pattern of strabismus. Shaaban et al.^16^ analyzed 55 Japanese families in which at least 2 family members had either eso- or exodeviations. The authors concluded that the mode of inheritance was not compatible with conventional Mendelian inheritance. The results of our study are consistent with these two studies because most of our patients also demonstrate a non-Mendelian inheritance pattern.

Seeley et al.^20^ examined 48 patients from 33 families with familial AET, and compared 112 family members of these patients with a gender- and age-matched group of 20 AET patients with no known family history. The authors identified a pattern of inheritance in 8 families, of which 75% was autosomal recessive. Comparing the clinical characteristics of familial and non-familial AET patients revealed no difference in terms of refraction, stereopsis, or the likelihood of subsequent strabismus surgery.

Our pedigree analyses showed that 116 probands (54.0%) were related to at least 1 other strabismic individual. The positive rate of family history was 59% in the AET group, 45.5% in the PAET group, and 38.1% in the IET group. We found 1-2 affected family members in 35.1% of families, 3-4 affected members in 15.3% of families, and 5 or more affected members in 3.2% of families. When parents who underwent complete ophthalmic examinations were considered, 40.5% of probands in the AET group, 25.6% in the PAET group, and 23.1% in the IET group had an affected parent.

Birch et al.^21^ investigated 95 consecutive patients with esotropia, aged 18-60 months and obtained related data from a total of 2828 blood relatives. Overall, 22% of the study group was found to have 1 affected first-degree relative, 77% had first- and/or second-degree relatives, and 91% had at least 1 affected relative. By contrast, in our patient cohort, the familial occurrence rate of strabismus in first-degree relatives was 46%, while 56% had at least 1 affected relative. Therefore, the prevalence of strabismus in first-degree relatives in our study group was twice that observed by Birch et al.^21^, which might reflect the significant consanguinity rate in our study group.

Taira et al.^22^ studied a total of 327 Japanese strabismus patients, 101 with IET, 83 with AET or PAET, and 143 with intermittent XT. Each subject was evaluated for background factors such as family history, abnormalities during pregnancy, and any issues associated with delivery. A positive family history was detected in 22% of subjects in the IET group, 25% in AET and PAET groups, and 32% in the intermittent XT group. The positive rate of family history was similar for each type of comitant strabismus.

Mass screening for genetic eye diseases has been performed on more than 700000 people across numerous districts in China to investigate the prevalence and mode of inheritance of major genetic eye diseases. More than 5000 pedigrees with genetic eye diseases were evaluated. Among these, Hu^23^ investigated the mode of inheritance of XT in 425 individuals. The familial occurrence rate in first-, second-, and third-degree relatives was 9%, 2.2%, and 1.1%, respectively. Moreover, the prevalence of XT was 0.58% and the heritability was calculated as 81.3%.

In Sweden, Abrahamsson et al.^24^ followed 1571 children with a reported family history of strabismus for 6 years from the age of 1 year. They found that a positive family history of strabismus led to a 3-fold increase in the risk of developing strabismus. In cases with 2 strabismic parents, the risk was increased to 7-fold. Ziakas et al.^25^ conducted a study on 96 probands with IET, AET, anisometropic esotropia, and XT. A complete 3-generation pedigree was established for each subject. From a total of 2074 family members, 67.3% of 49 cases in the AET group had at least 1 first-degree relative affected with strabismus, although the subtype was not specified; this percentage was significantly higher than in the other three groups.

In our study, the likelihood that a subject had a strabismic sibling was 29.3% in the AET group, 23.1% in the PAET group, and 30.8% in the IET group. Similarly, Richter^12^ found that the incidence of strabismus or strabismus-associated ocular anomalies among siblings of an affected proband was 20% if both parents were unaffected, and 30%-40% if one or both parents were affected. Chimonidou et al.^13^ examined 345 affected brothers and sisters having comitant strabismus who originated from 170 families. The frequency of congenital strabismus (strabismus within the first year of life) was 42.9% (n=148). Out of 148 patients with congenital strabismus, 42% had a sibling affected at a more advanced age, while the remaining patients were brothers and sisters who developed strabismus at the same age. In 96.5% of siblings, strabismus was concordant. Ferreira et al.^26^ ophthalmically evaluated 110 strabismic probands from 107 families and 329 associated relatives, observing a high prevalence of strabismus within each family, although the type of deviation varied between individuals. Almost half (46%) of the families with more than 1 affected individual included both exotropes and esotropes, suggesting an autosomal dominant inheritance pattern.

The frequency of microtropia among proband mothers was 14.7% in the AET group, 12.8% in the PAET group, and 14.7% in the IET group of the present study. This compared with frequencies of 14.7% in the AET group but only 2.7% in the PAET group among proband fathers. The higher prevalence of microtropia in mothers compared with probands fathers may be related with the high prevalence of anisometropia found in proband mothers. Close association between microtropia and anisometropia has been supported by previous studies.^4,8^ Cantolino and von Noorden^4^ found close associations between microtropia and large-angle strabismus in other family members. They suggested that because of the high incidence of binocular vision abnormalities observed in family members of microtropia patients, microtropia was not a segregating phenotype but was rather caused by multifactorial inheritance. Scott et al.^8^ reported a 7.7% prevalence of primary monofixation syndrome among family members of IET patients, which is higher than the 1% observed for the general population. They suggested that primary monofixation syndrome represents the partial expression of a genotype that codes for esotropia.

In our study, the incidence of hypermetropia ≥+3.00 D was similar for all strabismic subtypes. The frequency of hypermetropia ≥+3.00 D among first-degree relatives was 16.3% in the AET group, 5.2% in the PAET group, and 7.7% in the IET group. Hypermetropia ≥+3.00 D was not found in IET or PAET siblings, but was observed in 11.2% of AET siblings. The frequency of hypermetropia ≥ +3.00 D among first-degree relatives with strabismus differed significantly among groups, at 36.7% for the AET group, 13.3% for the PAET group, and 25% for the IET group. Shah et al.^27^ prospectively examined 81 probands with AET and their 115 siblings for the prevalence of amblyogenic risk factors, and found that 14.8% had strabismus and 23.5% had hypermetropia ≥+3.50 D.

### Study Limitations

Our study group consists only of a Turkish population without ethnic heterogeneity and having a considerable rate of consanguinity. This increases the statistical power of our investigation of the role of recessive inheritance in strabismus. Additional studies of families with multiple affected members should be conducted to identify the genetic mechanism underlying comitant strabismus.

## Conclusion

Although the gene(s) responsible for comitant strabismus remain to be identified, the genetic etiology of this condition is indisputable. The results of our study support the concept that a positive family history significantly increases the risk of a strabismic deviation, which was shown to be independent of refractive error heritability; this was especially true for AET. We also found that the autosomal recessive mode of inheritance was not a frequent pattern of inheritance, even in the presence of consanguinity.

## Figures and Tables

**Table 1:**
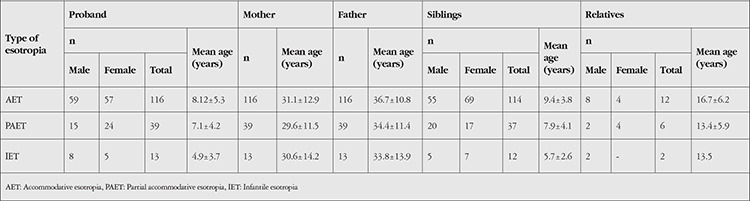
Demographic characteristics of probands and family members who underwent ophthalmic examination

**Table 2:**
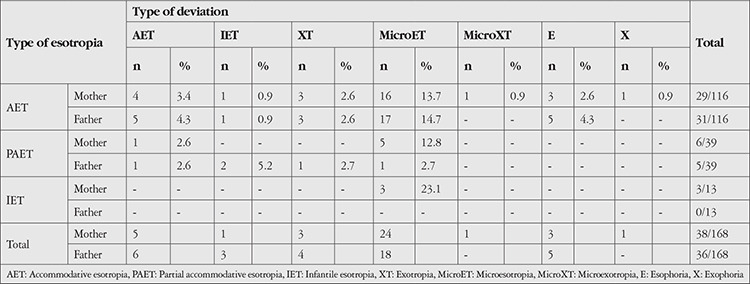
Strabismus prevalence and types of strabismus found in parents by physical examination

**Table 3:**
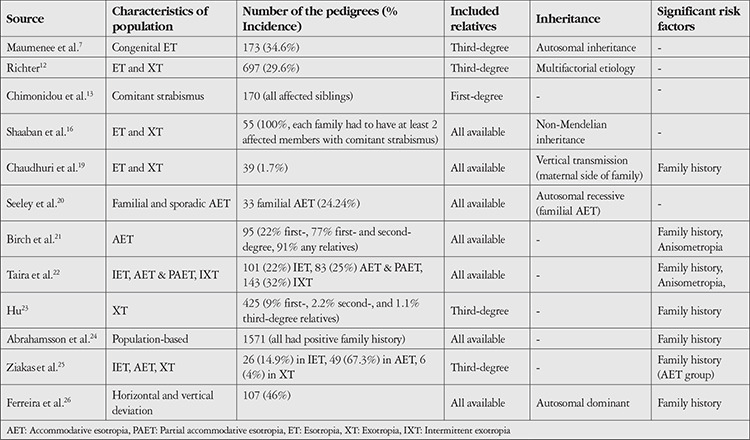
Characteristics and risk factors assessed in family and population studies
